# Supplementary material for: Sponge bHLH Gene Expression in Xenopus laevis Disrupts Inner Ear and Lateral Line Neurosensory Development and Otic Afferent Pathfinding
Source: Int J Mol Sci. 2025 Jun 7;26(12):5487. doi: 10.3390/ijms26125487 (PMC12193494; doi:10.3390/ijms26125487)

Supplemental Figure S1. 500pg AmqbHLH1 mRNA severely disrupts developmental processes. Compared with a control *Xenoups laevis* stage 46 tadpole (A), shows the normal formation with an ear (B) while there is near on the right ear (B'). Scale bar is 1mm.

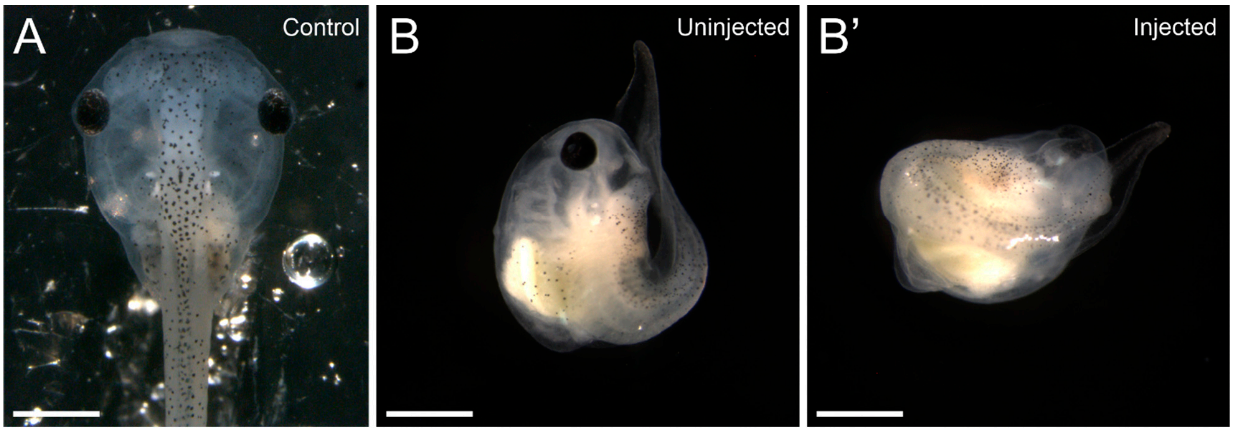

Supplement: Supplementary file 1 [file ijms-26-05487-s001.zip › ijms-3659085-supplementary.pdf]
